# A new modulated crystal structure of the ANS complex of the St John’s wort Hyp-1 protein with 36 protein molecules in the asymmetric unit of the supercell

**DOI:** 10.1107/S2059798320006841

**Published:** 2020-06-17

**Authors:** Joanna Smietanska, Joanna Sliwiak, Miroslaw Gilski, Zbigniew Dauter, Radoslaw Strzalka, Janusz Wolny, Mariusz Jaskolski

**Affiliations:** a Faculty of Physics and Applied Computer Science, AGH University of Science and Technology, Krakow, Poland; b Center for Biocrystallographic Research, Institute of Bioorganic Chemistry, Polish Academy of Sciences, Poznan, Poland; c Department of Crystallography, Faculty of Chemistry, A. Mickiewicz University, Poznan, Poland; d Synchrotron Radiation Research Section, National Cancer Institute, Argonne National Laboratory, Argonne, IL 60439, USA

**Keywords:** superstructure modulation, *Hypericum perforatum*, Hyp-1

## Abstract

Superstructure modulation, with violation of the strict short-range periodic order of consecutive crystal unit cells, is well known in small-molecule crystallography but is rarely reported for macromolecular crystals. To date, one modulated macromolecular crystal structure has been successfully determined and refined for a pathogenesis-related class 10 protein from *Hypericum perforatum* (Hyp-1) crystallized as a complex with 8-anilinonaphthalene-1-sulfonate (ANS) [Sliwiak *et al.* (2015 ▸), *Acta Cryst.* D**71**, 829–843]. The commensurate modulation in that case was interpreted in a supercell with sevenfold expansion along **c**. When crystallized in the additional presence of melatonin, the Hyp-1–ANS complex formed crystals with a different pattern of structure modulation, in which the supercell shows a ninefold expansion of **c**, manifested in the diffraction pattern by a wave of reflection-intensity modulation with crests at *l* = 9*n* and *l* = 9*n* ± 4. Despite complicated tetartohedral twinning, the structure has been successfully determined and refined to 2.3 Å resolution using a description in a ninefold-expanded supercell, with 36 independent Hyp-1 chains and 156 ANS ligands populating the three internal (95 ligands) and five interstitial (61 ligands) binding sites. The commensurate superstructures and ligand-binding sites of the two crystal structures are compared, with a discussion of the effect of melatonin on the co-crystallization process.

## Abstract 2

When crystallized in complex with the fluorescent dye 8-anilinonaphthalene-1-sulfonate (ANS) in the presence of melatonin, Hyp-1, a pathogenesis-related class 10 protein from *Hypericum perforatum*, produced tetartohedrally twinned *C*2 crystals with commensurate structure modulation, which was interpreted as a ninefold expansion of the unit cell in the **c** direction. The asymmetric unit of this supercell contains 36 protein molecules (differently populated by 156 ANS ligands) arranged into columns by a combination of ninefold translational noncrystallographic symmetry and pseudotetragonal rotational NCS.

## 1. Introduction   

Crystal structure modulation (also known as superstructure) is classified with other aperiodic phenomena as it consists of the violation of short-range unit-cell-to-unit-cell periodicity, which over the long range is regained by a wave of structural deformations, described by an atomic modulation function (AMF). In reciprocal space, this structural phenomenon is manifested by the presence of main reflections (usually stronger but also affected by the modulation) arising from the (approximately periodic) main lattice of the basic unit cells and of (usually weaker) satellite reflections arising from the periodic AMF. Since in real space the period of the AMF is longer than the corresponding period of the basic lattice, in reciprocal space the satellites subdivide the wider main-to-main distances. The situation is relatively simple, and the phenomenon is termed commensurate modulation, if the period of the AMF is an integral multiple of the corresponding period of the basic lattice. The structure may be then described, not quite elegantly but efficiently, in an expanded unit cell called a supercell. In the more general incommensurate case such a trick is not possible and the mathematical solution lies in the concept of superspace, as introduced by Janner, Janssen and de Wolff (Janner & Janssen, 1977 ▸; de Wolff, 1974 ▸), which makes explicit use of the AMF function. The phenomenon of superstructure modulation has been relatively well studied in small-molecule crystallography (Schönleber, 2011 ▸), but is very rarely mentioned in the context of macromolecular crystals. Superspace theory and its application by Sander van Smaalen (van Smaalen, 2004 ▸, 2007 ▸) inspired work on symmetry analysis of the diffraction from (3 + 1)-dimensionally incommensurately modulated crystals of profilin–actin (PA; Lovelace *et al.*, 2004 ▸; Porta *et al.*, 2011 ▸). The solution and refinement of 11 twinned and incommensurately modulated structures of engineered variants of the *Escherichia coli* enzyme *N*-acetylneuraminic acid lyase have been reported by Ivan Campeotto (Campeotto *et al.*, 2018 ▸). Recent years brought promising results in assigning a (3 + 1)-dimensional superspace group to an incommensurately modulated protein crystal (Porta *et al.*, 2017 ▸), as well as in solving and refining a model structure using a supercell approximation (Lovelace *et al.*, 2013 ▸, 2019 ▸). Jeffrey Lovelace and Gloria Borgstahl have given an excellent overview of the available methods for solving problematic macromolecular crystal structures marked by order–disorder phenomena and positional modulation (Lovelace & Borgstahl, 2020 ▸).

To the best of our knowledge, however, a complete successful macromolecular crystal structure determination has only been reported for the pathogenesis-related class 10 (PR-10) protein Hyp-1 from St John’s wort (*Hypericum perforatum*) which, when crystallized after pre-incubation with 8-anilinonaphthalene-1-sulfonate (ANS), produced tetartohedrally twinned crystals with sevenfold modulation along the **c** cell axis (Sliwiak *et al.*, 2014 ▸, 2015 ▸). The superstructure of this Hyp-1–ANS crystal (hereafter referred to as 7Hyp/ANS) was solved in space group *P*1 by a version of *Phaser* (McCoy *et al.*, 2007 ▸) that included translational noncrystallographic symmetry (tNCS) targets in the molecular-replacement algorithm (Sliwiak *et al.*, 2014 ▸), and was then refined in the correct *C*2 space group using *REFMAC* (Murshudov *et al.*, 2011 ▸) with the twin option switched on. In other words, the whole structure-determination process (additionally exacerbated by complex twinning) was handled using a commensurate model in a supercell with sevenfold expansion of the **c** parameter. Since the basic structural motif of 7Hyp/ANS consisted of a pair of Hyp-1 dimers, the asymmetric part of the supercell comprised 28 protein molecules supplemented by 89 ANS sites. After detailed analysis of this structure, it was concluded that it was the atypical pattern of docking of the ANS ligands within and around the 28 protein chains that was the driving force for the modulation pattern (Sliwiak *et al.*, 2015 ▸).

For reasons that are explained below, in the present project we repeated the crystallization of Hyp-1 in the presence of a mixture of ANS and melatonin (MEL), and obtained another modulated phase of the Hyp-1–ANS complex in which a similar basic structural motif is repeated along the **c** axis not seven but nine times (hereafter referred to as 9Hyp/ANS). Except for the modulation pattern (which is clearly visible in the diffraction data), the structural symmetry (*C*2) is preserved, as is the physical tetartohedral twinning of the crystals. It is thus very intriguing why the modulation, which is viewed as a pathological deviation from regular crystal period­icity, becomes even more pathological on what would appear to be an insignificant tweaking of the crystallization conditions, and in a way that, while numerologically pleasing (a switch from 7 to 9), does not have a simple rational explanation. For example, the observations made so far could prompt the questions ‘are modulations ruled by the numbers 5 or 11 *etc.* also possible?’ or ‘are even numbers (6, 8, 10 *etc.*) in the modulation rule also possible?’.

PR-10 refers to a multi-gene family of plant-specific pathogenesis-related proteins of class 10 that are differentially expressed by plants under stressful conditions such as drought, salinity or pathogen invasion (Fernandes *et al.*, 2013 ▸). After much debate in the literature about the biological function of these abundant proteins, a consensus seems to be converging on their role in the binding and transport of metabolic mediators such as phytohormones, lipids or flavonoids. Such a ‘molecular container’ view is consistent with the canonical PR-10 fold, which is built around an internal void (cavity) formed by a seven-stranded antiparallel β-sheet curved in a gripping shape around a long C-terminal helix α3 (Gajhede *et al.*, 1996 ▸; Biesiadka *et al.*, 2002 ▸; Pasternak *et al.*, 2006 ▸). The seven β-strands form a progression connected by β-turns and loops L1–L9, except for strands β1 and β2 at the edges of the β-sheet, which are connected by a V-shaped fork of two short α-helices (α1 and α2) that acts as a support for the hovering helix α3. Loop L9 connects the N-terminal end of helix α3 to the protein scaffold. Access to the hydrophobic cavity is gated by two entrances: E1, which is shaped by loops L3, L5 and L7 and by the N-terminal end of helix α3, and E2, which runs as a crevice between the full length of helix α3 and strand β1. In a number of crystallographic studies the hollow core of PR-10 proteins, which is quite robust and spacious (Chwastyk *et al.*, 2014 ▸, 2016 ▸), has been reported as the docking site for bio­logical ligands such as the phytohormones cytokinins (Pasternak *et al.*, 2006 ▸; Fernandes *et al.*, 2008 ▸; Ruszkowski *et al.*, 2013 ▸; Sliwiak *et al.*, 2016 ▸), gibberellins (Ruszkowski *et al.*, 2014 ▸) or abscisic acid (Sheard & Zheng, 2009 ▸). Most recently, the repertoire of plant hormones bound by PR-10 proteins has been extended by melatonin, which was first reported to form complexes with Hyp-1 (Sliwiak *et al.*, 2016 ▸) and subsequently with the yellow lupin protein LlPR-10.2B (Sliwiak *et al.*, 2018 ▸). The Hyp-1 protein is important in this context because it has a unique set of three ligand-binding sites consisting of two internal but separated chambers (sites 1 and 2) and a pocket formed by a deep surface invagination (site 3).

The biophysical characterization of complexes of PR-10 proteins with biological ligands is often difficult (for example because of solubility issues), and the ANS displacement assay (ADA; Gasymov & Glasgow, 2007 ▸) is frequently used as a method of choice to monitor the shift of ANS fluorescence on competition with the ligand of interest. The molecular basis of the ADA method was the primary motivation that prompted our crystallographic investigations of the Hyp-1–ANS complex. The first structure (Sliwiak *et al.*, 2015 ▸) showed an ANS-binding landscape at sites 1, 2 and 3 consistent with that found for the natural ligand melatonin, plus additional superficial binding sites where ANS molecules glue together neighboring protein molecules in the crystal. As mentioned above, the 7Hyp/ANS crystal revealed a case of macromolecular crystal structure modulation. In the present project, the idea was to test whether there was direct competition between ANS and melatonin for Hyp-1 binding. Accordingly, the protein was crystallized after incubation with a cocktail of ANS and MEL. The new 9Hyp/ANS crystals turned out to provide only meager direct evidence for the ability of melatonin to compete with ANS for Hyp-1 binding sites, but instead revealed a structure with an even more complex pattern of modulation.

## 2. Materials and methods   

### 2.1. Protein-complex formation and crystallization   

**Figure 1:**
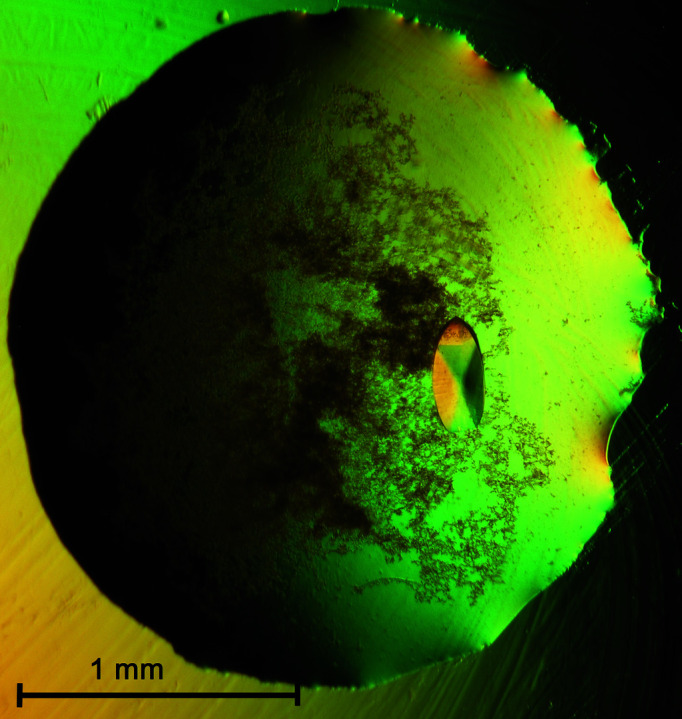
A single crystal of Hyp-1 protein (16 mg ml^−1^) co-crystallized with ANS (9 m*M*) and MEL (4.5 m*M*) using 0.1 *M* HEPES pH 7.5 buffer and 1.4 *M* sodium citrate as the precipitating agent. The hanging drop was mixed as a 1:1 volume ratio of the protein and reservoir solutions.

Hyp-1 was produced as described previously (Sliwiak *et al.*, 2015 ▸). Prior to crystallization, the protein solution was concentrated to 16 mg ml^−1^ and incubated simultaneously (competitively) with ANS and MEL for 1 h at 292 K. The protein:ANS molar ratio in the incubation mixture was 1:10, whereas the ANS:MEL ratio was 2:1, 1:1, 1:2 or 1:3. Crystallization trials for all four protein–ligand combinations were prepared with a 1.6–1.1 *M* gradient of sodium citrate as the precipitating agent using the conditions established previously for the crystallization of Hyp-1 complexes with MEL (1.3 *M* citrate buffer pH 6.3; Sliwiak *et al.*, 2016 ▸) or with ANS (0.1 *M* HEPES buffer pH 7.5; Sliwiak *et al.*, 2015 ▸). Crystals were only found in the latter case (‘ANS’ conditions with HEPES buffer) with a 2:1 ANS:MEL ratio and at the following sodium citrate concentrations: 1.6 *M* (after two days), 1.5 *M* (one day), 1.4 *M* (one week) and 1.3 *M* (two months). For drops mixed with a 2:1 protein:reservoir volume ratio, the rod-like crystals were reminiscent of the previously reported crystals of the 7Hyp/ANS complex (Sliwiak *et al.*, 2015 ▸) but only diffracted to ∼3.0 Å resolution. When the protein:reservoir volume ratio was 1:1, lens-shaped crystals appeared (Fig. 1 ▸) that diffracted X-rays to 2.3 Å resolution.

### 2.2. Data collection and processing   

X-ray diffraction data were collected on beamline 14.2 at the BESSY II synchrotron (Table 1 ▸). The data were processed to 2.3 Å resolution with *HKL*-3000 (Minor *et al.*, 2006 ▸) and scaled in triclinic symmetry with an *R*_merge_ of ∼0.14. The *a* and *b* parameters of the unit cell were equal and roughly the same as for the previously characterized 7Hyp/ANS crystal (Sliwiak *et al.*, 2015 ▸), but the *c* parameter of ∼386 Å was ∼9/7 times longer than that in 7Hyp/ANS. This suggested even at this early stage that the present crystal might have a modulated superstructure with nine rather than seven structural motifs repeated in the **c** direction.

### 2.3. Structure solution   

Since the true space group of the structure was unknown at this point because of apparent twinning, structure solution by molecular replacement (MR) was attempted in the triclinic space group *P*1. The molecular model for MR, consisting of 36 Hyp-1 molecules, was prepared starting from the contents of the asymmetric unit of the 7Hyp/ANS structure (seven repeats of a molecular tetrad: *AB*–*ab*, *CD*–*YZ*, *EF*–*WX*, …, *MN*–*OP*) and extending it at the *MN*–*OP* end by **c**-translated copies of *AB*–*ab* and *CD*–*YZ*.

The *Phaser* program (McCoy *et al.*, 2007 ▸) found two copies of this probe (*i.e.* 72 Hyp-1 molecules) in the triclinic unit cell. A quick refinement of this model in *REFMAC* reduced the *R* factor to ∼0.25 and automatically detected eight twin domains. In real space, this solution corresponded to a *C*-centered lattice with monoclinic symmetry, as in the 7Hyp/ANS case. Accordingly, a nonredundant model was selected composed of one half, *i.e.* 36 protein molecules, of the *P*1 solution. Because of the extra symmetry of the *C*2 space group, the apparent eightfold twinning was reduced to fourfold, *i.e.* to tetartohedry, again in analogy to the 7Hyp/ANS case.

### 2.4. Structure refinement   

A set of 3974 *R*_free_ reflections were selected using *SHELXPRO* (Sheldrick, 2015 ▸) from narrow reflection shells to ensure the inclusion of NCS-related and twin-related reflections. The structure was refined in *REFMAC*5 (Murshudov *et al.*, 2011 ▸) using intensity-based maximum-likelihood targets with twin detection/refinement and jelly-body algorithms. Only isotropic *B* factors were applied. Bulk-solvent correction was introduced using Babinet’s scaling (Fenn *et al.*, 2010 ▸). Standard stereochemical restraints (Engh & Huber, 1991 ▸) were used for the protein molecules, whereas the geometrical restraints for the ANS molecules were created using the coordinates of the ammonium salt of ANS found in the Cambridge Structural Database (Groom *et al.*, 2016 ▸) under the identifier CSD-AMANNS (Weber & Tulinsky, 1980 ▸) and the *Mercury* software package (Macrae *et al.*, 2020 ▸). Weights for restraining bonds, angles and planarity were defined as standard deviations of 0.01 Å, 3° and 0.01 Å, respectively. AMANNS (*R* value 3.5%) was considered to be a better CSD model for ANS than the ANAPHS structure (Cody & Hazel, 1977 ▸; *R* = 6.8%) used for ANS restraints in the case of 7Hyp/ANS.

## 3. Results and discussion   

### 3.1. Modulation in reciprocal and direct space   

**Figure 2:**
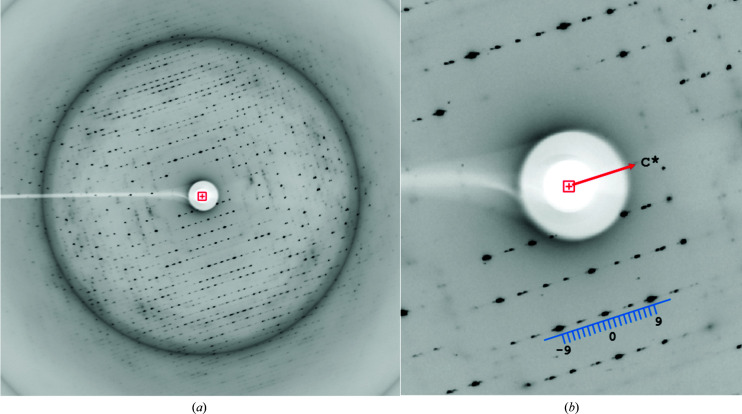
X-ray diffraction images of the 9Hyp/ANS crystal. (*a*) A full diffraction image. The ice ring corresponds to 3.3 Å resolution. (*b*) Enlargement with main (−9, 0, 9) and satellite (other marks) reflections marked (as the index *l*) along the c* direction.

**Figure 3:**
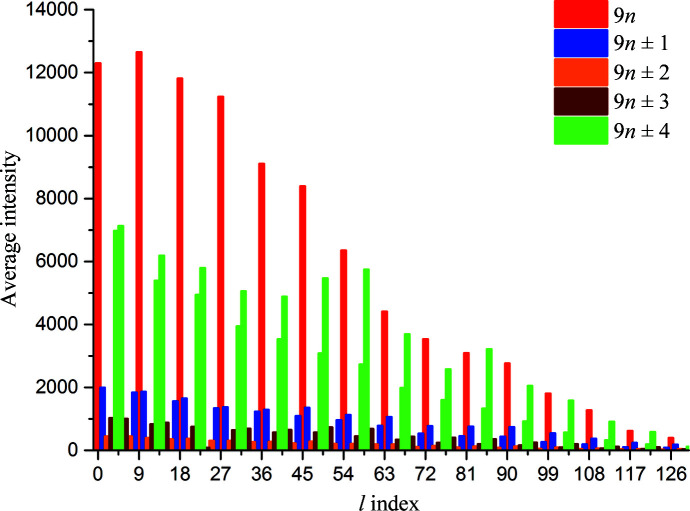
Plot of average reflection intensity for layers of constant *l* index. *l* = 9*n* layers are shown in red and *l* = 9*n* ± 4 layers in green.

In reciprocal space, 9Hyp/ANS modulation is manifested by a unique diffraction pattern consisting of stronger main Bragg peaks flanked by weaker satellite reflections, in analogy to the pattern reported for 7Hyp/ANS (Fig. 2 ▸). Also, analysis of the intensity modulation in shells of constant *l* index reveals a pattern based on the same principle as that for 7Hyp/ANS, but with the strongest intensities spaced regularly in reciprocal space at *l*-index intervals of 4.5 (Fig. 3 ▸). This distribution is based on indexing the diffraction pattern using the expanded supercell (encompassing the entire wave of modulation in the **c** direction); in such an approach the main and satellite reflections are indexed (*l*) consecutively along c*, without any subdivision. In this description, the modulation in real space is treated as very extensive (ninefold) tNCS. The presence of this tNCS was also evident from very strong 0, 0, *n*/9 non-origin peaks in the native Patterson, with a maximum (70% of the origin peak) at *w* = 2/9 (not shown).

### 3.2. Twinning analysis   

The presence of crystal twinning affects the deviation of the reflection intensities from the theoretical Wilson distribution owing to the averaging of disproportionate fractions of very strong and very weak reflections (Yeates, 1988 ▸). Unfortunately, twinning has the opposite statistical effect (a low proportion of very weak reflections) to tNCS (many very weak reflections), making twin detection for crystal structures with tNCS problematic or even impossible.

**Figure 4:**
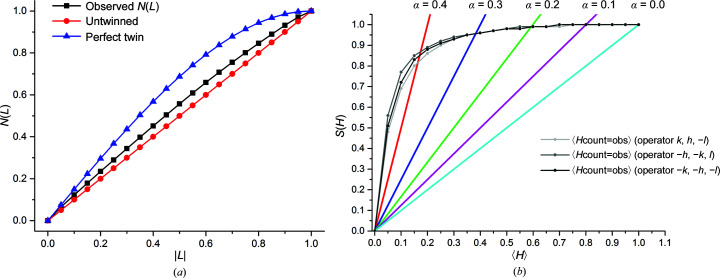
(*a*) Cumulative distribution of *N*(*L*) (*L*-test) calculated in *TRUNCATE* (French & Wilson, 1978 ▸) for perfectly twinned and untwinned data in comparison with the experimental diffraction data for 9Hyp/ANS. The blue and red curves represent the expected cumulative distributions of |*L*| for perfectly twinned and untwinned data, respectively, for acentric reflections. The black line shows the calculated cumulative distribution for the 9Hyp/ANS acentric diffraction data, indicating at least partial twinning. (*b*) Cumulative distribution of the *H* ratio [*S*(*H*) test] for acentric reflections of the 9Hyp/ANS diffraction data set. Colored continuous lines indicate theoretical cumulative distributions for twin fractions (α) of 0.0–0.4. The gray dotted lines show *S*(*H*) for (*k*, *h*, −*l*), (−*h*, −*k*, *l*) and (−*k*, −*h*, −*l*) subsets of reflections paired with (*h*, *k*, *l*). The shape of the experimental distributions suggests a twin fraction of ∼0.45 for each twin operator.

Twin operators and fractions for 9Hyp/ANS were estimated from the cumulative *H* ratio distribution for pairs of twin-related intensities. The calculated twin fractions (nearly 0.45 in the *H*-test for each detected twin operator) might be overestimated relative to the values of 0.25 expected for perfect tetartohedry owing to the presence of rotational NCS in direct space that almost coincides with the potential twin operators (Roversi *et al.*, 2012 ▸). Detection of twinning was also carried out using the *L*-test (Padilla & Yeates, 2003 ▸), in which reflections locally related in reciprocal space are selected to calculate the cumulative |*L*| ratio distribution. The results of the *L*-test for data merged in the correct monoclinic space group (〈|*L*|〉 = 0.459 and 〈*L* ^2^〉 = 0.290) indicate at least partial twinning (Fig. 4 ▸ *a*). The advantage of the *L*-test over the *H*-test is its insensitivity to rotational and (with an appropriate selection of *l*-index difference, here 9) also to translational NCS. Other crystallographic pathologies present in 9Hyp/ANS, such as tNCS, can mask the effect of twinning by reducing the intensity of whole subsets of reflections, which leads to intensity modulations in index groups and additional deviations, usually opposite to those arising from twinning, from the expected intensity distribution (Clifton *et al.*, 2017 ▸).

### 3.3. Refinement statistics and final model quality   

The 9Hyp/ANS model contains 36 independent protein molecules in the asymmetric unit, labeled *A*, *B*, …, *Z*, *a*, *b*, …, *i*, *j*. The initial electron-density maps were of poor quality, with gaps in the protein main chains and blurred electron density for many side chains. After several rounds of manual rebuilding in *Coot* (Emsley *et al.*, 2010 ▸) interspersed with automatic *REFMAC* refinement, the final electron-density maps allowed the tracing of all 36 protein chains without breaks. Moreover, most of the side chains have clear definition, except for several Phe(−1) and Met1 side chains at the N-termini of the protein chains, which were excluded from the model.

Refinement of the 9Hyp/ANS structure with data extending to 2.3 Å resolution and with isotropic atomic displacement parameters (ADPs) converged at an *R*_work_ and *R*_free_ of 0.226 and 0.257, respectively (Table 2 ▸). An attempt to model anisotropic motion/disorder with TLS parameters did not improve the *R*-factor statistics. The good quality of the final model is confirmed by the well ordered trace of the C-terminal helix α3 (which is often disordered or modeled in poor electron density in PR-10 structures) and the mostly clear appearance of side-chain electron density, allowing the unambiguous identification of rotamers. As assessed by *MolProbity* (Chen *et al.*, 2010 ▸), 92.6% of the main-chain φ/ψ torsion angles are located in the most favored areas of the Ramachandran plot, with only 0.5% as outliers. Most of the violations of polypeptide backbone conformation occur in the area of four external loops (L4, L7, L8 and L9), particularly at Val60 and Glu131, the side chains of which are involved in multiple intermolecular contacts in several protein molecules.

A careful examination of difference electron-density maps revealed the location of 156 ANS molecules as well as 22 other moieties from the crystallization solution. Near entrance E1 of protein chain *V*, a large patch of electron density was observed with *mF*_o_ − *DF*_c_ peaks as high as 11.3σ. It was tentatively interpreted as a melatonin (MEL) ligand, which refined with a high average *B* factor (91.5 Å^2^). Further analysis using polder OMIT maps as implemented in *Phenix* (Liebschner *et al.*, 2019 ▸) did not confirm this interpretation, with a map–model correlation coefficient equal to 0.49. Ultimately, this area was marked with five water molecules labeled as an unidentified ligand (UNL). Its location differs from the location of MEL molecules in the Hyp-1–MEL complex, which were identified only in the internal cavity of the protein and in its surface invagination (Sliwiak *et al.*, 2018 ▸).

The final model includes ten sulfate anions, which can be considered as visible remnants of ANS sulfonate groups or as independent sulfate/phosphate ions. A single fully occupied HEPES (EPE) molecule was found interacting with residues near entrance E1 of chain *P* at a binding position normally reserved for ANS. The model also includes seven citrate anions (FLC), which were used in the crystallization buffer as precipitant, and three dimethyl sulfoxide (DMS) molecules from the ANS stock solution.

### 3.4. Hyp-1 dimer formation   

In general, PR-10 proteins such as Hyp-1 are monomeric under physiological conditions and in their hypothetical ligand-binding function, but there are notable exceptions. In particular, the *Medicago truncatula* nodulin MtN13 with the PR-10 fold forms dimers by reciprocal intermolecular docking of the Asn79 side chain in the cytokinin-filled binding cavity (Ruszkowski *et al.*, 2013 ▸). Hyp-1 itself, when crystallized under oxidizing conditions as a serendipitous complex with PEG, turned out to exist as a covalent dimer formed via an intermolecular S—S bridge involving the sole Cys126 residue in its sequence, as well as a β-sheet dimer formed by parallel association of the β1 strands with another Hyp-1 partner molecule (Michalska *et al.*, 2010 ▸). Also, in the 7Hyp/ANS structure the protein molecules formed side-by-side dimers as a consequence of an intermolecular antiparallel β1–β1 sheet interaction (Sliwiak *et al.*, 2015 ▸). A similar association is observed in the present structure, where dimers are created by antiparallel association of the β1 strands of adjacent protein molecules.

**Figure 5:**
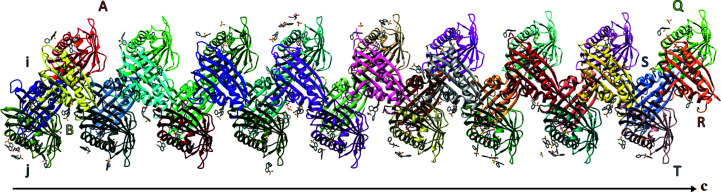
Overall packing of the 36 Hyp-1 molecules (labeled *A*, *B*, …, *Z*, *a*, *b*, …, *j*) in the asymmetric part of the expanded unit cell. The protein molecules are arranged with ninefold noncrystallographic repetition of the same structural motif (two Hyp-1 dimers, for example *AB* and *ij*) along **c** (black arrow). Small-molecule ligands are shown in ball-and-stick representation.

Analogously to the 7Hyp/ANS structure, the protein chains in the 9Hyp/ANS crystal are arranged into 18 β1–β1 dimers labeled *AB*, *CD*, …, *ij*. The dimers *AB*, …, *QR* have the same orientation and a pseudoperiodic repetition of 1/9 along **c**, leading to the creation of Row I. Row I is reproduced by the operation of a noncrystallographic 2_1_ screw axis along **c**, generating Row II consisting of dimers *ST*, …, *ij* (Fig. 5 ▸). The consecutive dimers across the pseudo-2_1_ axis are therefore related by an ∼180° rotation and a 1/18 translation along **c**. The curious screw that is thus formed could be annotated (by analogy to 7Hyp/ANS) as 2_1/9_.

There are some crystal-packing differences between 7Hyp/ANS and 9Hyp/ANS. The trivial difference is the presence of an additional eight protein molecules in the asymmetric unit, resulting in the elongation of the **c** axis from ∼298 to ∼385 Å and a ninefold repetition of the same motif in this direction. The 61 interstitial ANS molecules follow the helical pattern of the protein dimers in Rows I and II, but their distribution along the helix axis appears to be irregular and random.

### 3.5. Details of the pseudosymmetrical arrangement of the Hyp-1 molecules   

The characteristic crystal-packing scheme of Hyp-1 protein molecules along the longest unit-cell dimension combined with a large number of molecules in the asymmetric unit are hallmarks of crystal structure pseudosymmetry. The overall packing pattern comprising 36 protein molecules arranged into two columns (Rows) is shown in Fig. 5 ▸. The characteristic zigzag pattern of the two columns in the unit cell is the result of the noncrystallographic 2_1/9_ screw-axis operation, which combines an ∼180° rotation with an ∼1/18 translation along the screw axis in the [001] direction. This main noncrystallographic screw axis is crossed by two pseudotwofold axes aligned approximately with the [110] and [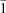
10] directions, which are the local dyads within and between the Hyp-1 dimers, respectively. The true crystallo­graphic twofold axis (as well as the true 2_1_ screw) runs in the [010] direction, perpendicular to the 2_1/9_ pseudo-screw and diagonally between the pseudodyads.

**Figure 6:**
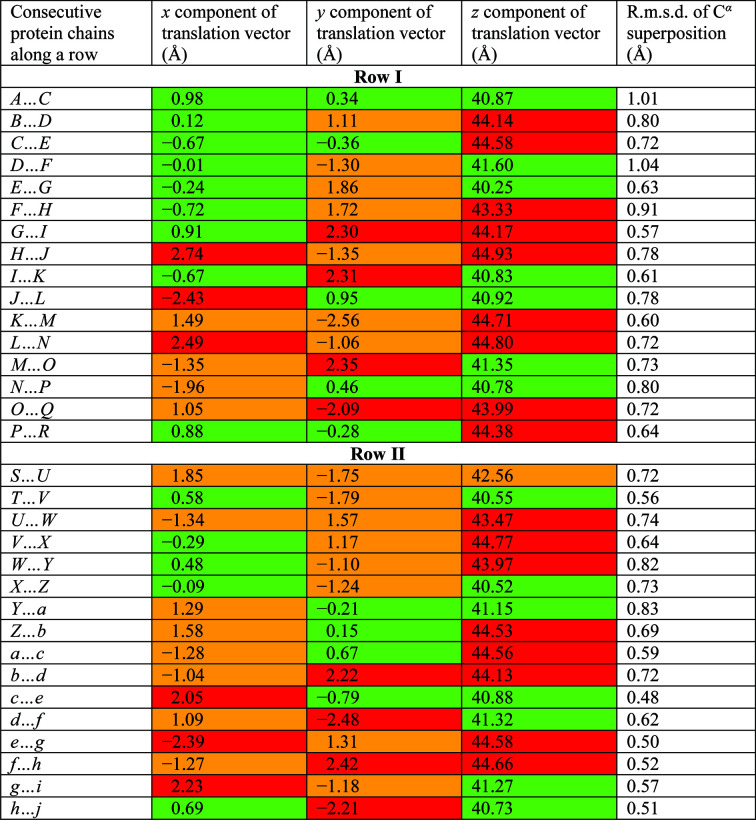
Perturbations of strict translational order along the crystallographic axes **a**, **b** and **c** of consecutive Hyp-1 molecules in Rows I and II, expressed in Å as components of the corresponding translation vectors. The translations were calculated for the C^α^ atom of Lys83, which has a stable and fixed position in the protein core. The violations (wavering) of strict translational order along **a** and **b** (expected to be 0 Å) are highlighted by color according to their severity as green (negligible perturbations, 0–1.0 Å), orange (small perturbations, 1.01–2.0 Å) and red (large perturbations, above 2.0 Å). Likewise, the **c** translations (expected to be 385.40 Å/9 = 42.82 Å) are marked in green (40.0–41.6 Å), orange (41.61–43.2 Å) and red (above 43.21 Å). R.m.s.d. values of C^α^ superpositions of the translated protein molecules are also shown in Å. All calculations were performed in *GESAMT* (Krissinel, 2012 ▸).

The translational tNCS component of the 2_1/9_ pseudo-screw has marked imperfections, which are visible as variations of the centroid–centroid distances between the consecutive Hyp-1 molecules along the Rows (Fig. 6 ▸) and minor distortions along the **a** and **b** crystallographic axes, as well as irregularities in ANS distribution within and between the protein chains. Nevertheless, the patterns of centroid–centroid distances and of ANS populations, which can be regarded as structural manifestations of the AMF function, are strictly reproduced after nine repetitions, which allows a description of this commensurate structural modulation in terms of an expanded supercell.

### 3.6. Comparison with the sevenfold-modulated superstructure of 7Hyp/ANS   

**Figure 7:**
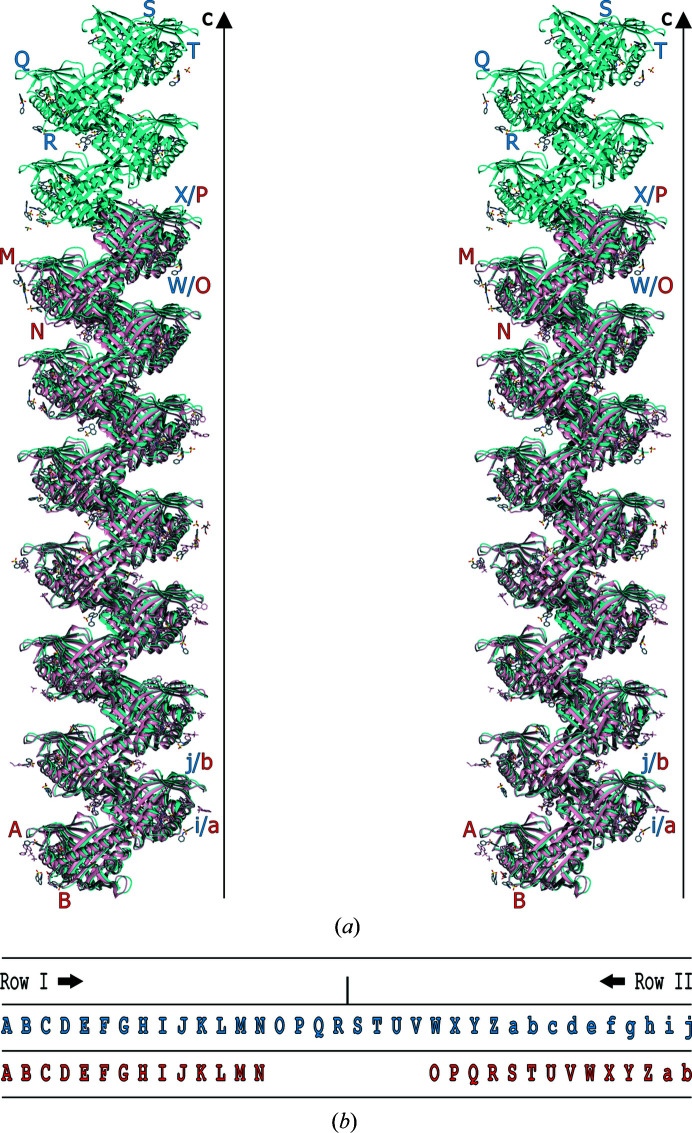
(*a*) Stereoview of superposition of the corresponding 9Hyp/ANS (blue) and 7Hyp/ANS (red) crystal-packing patterns in the asymmetric part of the expanded unit cells. The protein molecules are labeled *A*, *B*,…, *Z*, *a*, *b*, …, *j* and arranged along **c** (black arrow). The direction of labeling (along the **c** direction in Row I and against it in Row II) is preserved in both structures. The additional eight molecules in 9Hyp/ANS are appended at the end of Row I and the beginning of Row II of 7Hyp/ANS. Small-molecule ligands are shown in ball-and-stick representation. (*b*) Mapping of corresponding protein chain labels between 9Hyp/ANS (top, blue) and 7Hyp/ANS (bottom, red). The direction of labeling (along the **c** direction in Row I and against it in Row II) is preserved in both structures. The additional eight molecules in 9Hyp/ANS are attached at the end of Row I and the beginning of Row II of 7Hyp/ANS.

**Figure 8:**
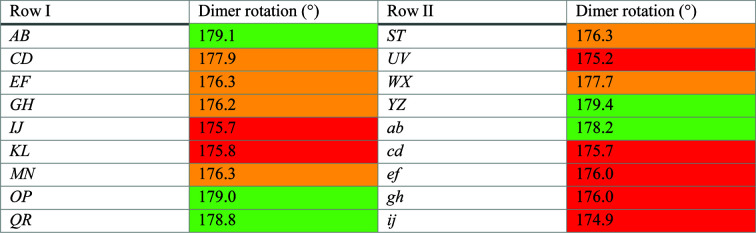
The degree of rotation (°) within the Hyp-1 dimers in Rows I and II, corresponding to rotational NCS. Rotations of between 178° and 180° are highlighted in green, those between 176.01° and 177.99° in orange and those of less than 176.01° in red. All calculations were carried out in *GESAMT* (Krissinel, 2012 ▸).

The crystal-packing arrangement of 9Hyp/ANS has a clear similarity to that known from the 7Hyp/ANS complex crystal structure (Fig. 7 ▸). Both patterns start at the same point of the unit cell along **c** (which in space group *C*2 is fixed by the twofold axes along **b**), so that division into Row I and Row II is preserved, the main difference being that the 9Hyp/ANS pattern is elongated by two molecular tetrads. The first seven dimers of the 9Hyp/ANS model building up Row I, *i.e.* *AB*, *CD*, …, *MN*, are directly superimposed on the same-labelled Hyp-1 molecules in Row I of 7Hyp/ANS when the origin and unit-cell axes of the two structures are aligned. The additional consecutive dimers of 9Hyp/ANS, *i.e.* *OP*, *QR* and *ST*, *UV*, form the end of Row I and the start of Row II, respectively. From dimer *MN*, which marks the last common block of the two labeling systems, the numbering of 9Hyp/ANS molecules is shifted by eight tags relative to that of 7Hyp/ANS (Fig. 3 ▸). The distances between the (centroids of) neighboring protein molecules in 9Hyp/ANS vary from 41 to 45 Å, which is comparable with the range of interdimer spacings within each Row of 7Hyp/ANS. While the protein dimers in both structures have approximate twofold rotational NCS, the degree of rotation differs significantly from the ideal 180° in 13 of the 18 9Hyp/ANS dimers (Fig. 8 ▸). A detailed analysis revealed perturbations (wavering) of the protein-molecule positions within the Rows along the crystallographic directions **a** and **b** ranging from 0.01 to 2.74 Å (the aberration in the **a** direction between chains *H* and *J* in Row I), which makes the 9Hyp/ANS crystal structure even more complex and unique (Fig. 6 ▸).

A massive C^α^-trace analysis of the polypeptide chains in the two structures did not reveal any major deviations between the protein molecules of these models or any patterns of increased/decreased similarities. The C^α^ r.m.s. deviations for chain-by-chain superpositions of the two models range from 0.47 Å (chains *H* and *c* from 7Hyp/ANS and 9Hyp/ANS, respectively) to 1.10 Å (chains *D* and *A*, respectively).

A more distinctive feature differentiating the two structures is the significantly larger number of ligand molecules in the 9Hyp/ANS complex. However, the population of the protein molecules by ANS ligands does not affect the main-chain conformation to any significant degree. For instance, within the 9Hyp/ANS structure the C^α^ r.m.s.d. for protein chains with drastically different ligand occupation (for example, chain *B* with seven out of eight binding sites fully occupied and chain *Y* with only one partially occupied ANS ligand) is only 0.68 Å, and this value is equal to the value calculated for the two equally occupied chains *B* and *F*. Furthermore, superposition of chain *V* (DMS and tentative MEL molecules bound in sites 6 and 7) on chains *f* (empty binding sites 6 and 7) and *d* (sites 6 and 7 fully occupied by ANS) reveals closer similarity between the C^α^ traces of chains *V* and *f* (0.56 Å) than between chains *V* and *d* (0.70 Å). This observation suggests that the binding of crystallization buffer components other than ANS (for example MEL, DMS or FLC) has little effect on the Hyp-1 framework conformation.

As a further highlight of the differences in protein–ligand population between the two structures, we note that in the 7Hyp/ANS structure there are two protein molecules (*T* and *V*) without any internal ANS ligands, whereas in 9Hyp/ANS there is at least one internal ligand in each Hyp-1 molecule (in chain *W* exactly one). Comparison of the C^α^ traces of chains *W* (9Hyp/ANS) and *T* (7Hyp/ANS) containing ANS only at two interstitial binding sites gives an r.m.s.d. of 0.54 Å.

### 3.7. Crystal lattice parameters   

With a switch from sevenfold to ninefold structure modulation exactly in the **c** direction, one would expect that the *c* lattice parameter should increase 9/7 times, from 298.56 to 383.86 Å. Yet the true *c* parameter of the 9Hyp/ANS crystal is 385.40 Å, *i.e.* longer than expected by 0.51%. This means that 9Hyp/ANS is not a simple elongated replica of the 7Hyp/ANS structure but that there must be a genuine structural difference.

Also, since the two structures are built on the same principle and appear to be isomorphous in the remaining two directions (**a**, **b**) of space, one would again expect that the *a* and *b* lattice parameters (fulfilling the serendipitous *a* = *b* relation in both cases) should have the same values in 7Hyp/ANS and 9Hyp/ANS. Yet, the *a* = *b* value in 9Hyp/ANS (148.85 Å) is larger by 1.75% than in 7Hyp/ANS (146.29 Å). This again points to a real, even if only slight, difference in the organization of the two structures.

Since all unit-cell parameters of 9Hyp/ANS are longer than expected from the application of the simple crystal-packing rules known from 7Hyp/ANS, one might suspect a miscalibration of the wavelength or goniometer geometry in one or both X-ray diffraction experiments. However, such miscalibrations, while known to exist, would not be expected to be of the order of 2%. Moreover, the two ‘corrective’ factors (0.51% and 1.75%) are significantly different, again suggesting a real structural, rather than an instrumental, difference. With this structural expansion of the 9Hyp/ANS unit cell (with the monoclinic β angle being exactly 90.00° in both cases), the Matthews volume of 3.12 Å^3^ Da^−1^ is slightly higher than that for 7Hyp/ANS (3.01 Å^3^ Da^−1^), corresponding to a slightly increased solvent content (60.6% *versus* 59.2%).

### 3.8. ANS binding sites   

The previous crystal structure of Hyp-1 in complex with ANS, 7Hyp/ANS, with its wealth of cases (28 protein molecules and 89 ANS sites) illustrating the protein–ligand interactions, allowed the identification of eight distinct ANS docking sites (1–8) and their further division into subgroups. The first group includes three specific internal binding sites 1–3, of which site 3 is a deep invagination of the protein surface and sites 1 and 2 are located within the general area of the PR-10 internal hydrophobic cavity. In Hyp-1 this internal cavity is divided into two distinct chambers, 1 and 2, with a separating partition (Arg27) and separate entrances, E1 and E2, respectively. Stabilization of ligand molecules in chamber 1 occurs mainly through hydrogen-bond formation between Arg27 and the sulfonate group of ANS. Hydrophobic residues such as Ala140 and Phe143 from helix α3 provide a hydrophobic environment for the aromatic core of ANS, while a system of additional polar tyrosine residues, Tyr84 (strand β5), Tyr101 (strand β6) and Tyr120 (strand β7), separates the ligand molecules trapped in the two inner chambers. Site 3 is created by stacking interactions, with the ligand molecule held in place between the jaws (Tyr150 from helix α3 and Lys33 from helix α2) of a molecular vise.

The remaining ANS binding sites 4–8 are interstitial, and are usually found to glue together the surfaces of three (or two in the case of sites 7 and 8) adjacent Hyp-1 molecules. The ANS ligands in the superficial sites 4, 5 and 6 concatenate adjacent protein chains into a unique helical pattern. The ANS molecules in sites 4 and 5 link three protein chains (two consecutive two chains from the same Row and a third one from the opposite Row) and are stabilized via hydrogen bonds between the ANS O atoms and the peptide group of Gly47 from loop L4 or Gly110 from loop L8. At site 5, the location of the ligand molecule is stabilized via hydrophobic interactions with Lys21 (from helix α1) and Ala77 (from loop L6). The ANS molecule anchored in site 6 can bind two or three protein chains depending on its position inside or on the periphery of the Row. The ANS molecules at sites 7 and 8, which are responsible for linking two adjacent protein chains, are anchored near entrances E1 and E2, respectively, and are stabilized by interactions with residues from loops L3 and L5. Table 3 ▸ presents a comparison of the amino-acid residues participating in protein–ANS interactions in the two modulated Hyp-1–ANS complex structures at the three internal binding sites 1–3.

#### 3.8.1. ANS occupation and comparison with 7Hyp/ANS   

**Figure 9:**
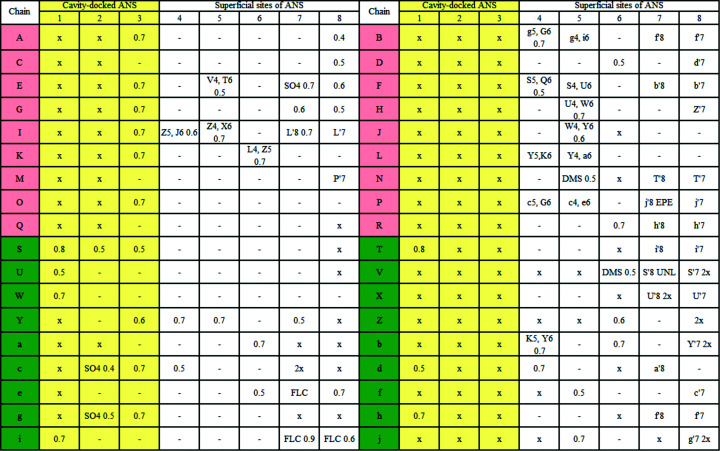
Scheme showing Hyp-1 saturation with ligand molecules in the crystal structure of 9Hyp/ANS. The 36 protein chains are grouped into two Rows, shown in pink (Row I; *AB*, …, *QR*) and green (Row II; *ST*, …, *ij*). The internal binding sites 1–3 are highlighted in yellow. Full ligand occupancy is marked by x or 2x to indicate the number of ligand molecules (one or two) found in the general area of the binding site. Partial occupancy is marked by the fractional value. For interstitial sites 4–8, the link to an adjacent protein molecule (and possible partial occupancy) is also indicated. The prime symbol (′) indicates symmetry-related molecules with respective binding sites. Ligand molecules differing from ANS (DMS, FLC, EPE and SO4) are denoted using their abbreviations and occupancies.

Although the ligand-binding sites are the same in the two complexes, the details of the ligand-saturation patterns within the asymmetric units are different. In the 9Hyp/ANS structure most of the ANS ligand molecules (95 of 156) are docked within the three internal binding sites 1–3, with 75 of them (78.9%) at full occupancy (Fig. 9 ▸). Furthermore, there are 61 ANS ligand molecules with very good electron density in the five additional surface binding sites 4–8. None of the protein chains is completely occupied by ligands at all eight sites or is completely unoccupied by ANS molecules. In dimers *AB*, …, *QR* forming Row I, one of the molecules (*A*, *C*, *E*, …, *Q*) has sites 1 and 2 always fully occupied, while its site 3 is either empty or partially filled, with ANS occupancy adjusted to 0.7. In the complementary molecules in these dimers (*B*, *D*, *F*, …, *R*) all three internal ANS binding sites 1–3 are always fully occupied with ligand molecules, which have excellent definition in the electron-density maps. The pattern in Row II is clearly different from the situation in 7Hyp/ANS, where many vacancies were registered in the first molecules of each dimer (*i.e.* *O*, *Q*, *S*, *U*, *W*, *Y*, *a*), and where site 3 of those molecules was always empty, while among the complementing molecules protein chains *T* and *V* had no internal ligands at all. In the case of 9Hyp/ANS, the second molecules (*T*, *V*, *X*, …, *j*) form a ligand-saturation pattern similar to Row I and always have a full set of ANS molecules in the three internal binding sites, with partial occupancy at sites *T*(1), *d*(1) and *h*(1). There are many perturbations in the ANS pattern in the first molecules of Row II (*S*, *U*, *W*, …, *i*), with three chains (*U*, *W*, *e*) containing only a single, partially occupied ligand molecule at site 1. Although site 1 is always occupied by ANS, site 2 of chains *c* and *g* is filled with a sulfate anion, which could be a component of the purification/crystallization buffer or, more likely, the only visible sulfonate signal from a poorly ordered ANS molecule.

In 7Hyp/ANS the ANS ligands were generally observed in their individual sites only at full occupancy, except for one partially occupied site 3 in chain *R*, whereas in 9Hyp/ANS there are as many as 50 ligand molecules (32.0% of all ANS sites) with partial occupancy. For instance, binding site 3 of the second molecule in both Rows is always empty or partially occupied. Taking into account all of the internal binding sites in 36 protein chains (36 × 3 = 108), it can be concluded that site 1 is always occupied, site 2 has five vacancies as well as two partially occupied sulfate anions instead of ANS, and site 3 is empty in eight cases. A similar inventory for 7Hyp/ANS shows that among 28 × 3 = 84 possibilities site 1 was empty in five cases, site 2 had three vacancies and site 3 was empty in 15 cases. The fact that in contrast to 7Hyp/ANS, where almost all ANS ligands are fully occupied, many ANS molecules have fractional occupancy in 9Hyp/ANS may be attributed to the competitive effect of melatonin during the crystal-growth process.

The saturation pattern of the superficial sites is much more complicated, with 61 surface-bound ANS molecules grouped into five available binding sites 4–8. An interesting observation is that in addition to the main ANS ligand, other crystallization components, such as EPE, DMS, FLC and possibly MEL, are also frequently found in the superficial sites. Overall, ten non-ANS molecules were identified in seven protein chains, occurring at the highest frequency at site 7 (five ligands). In chains *V* from Row II and *i* from Row I, two ligand molecules other than ANS were found to be simultaneously attached at sites 6 and 7, and at sites 7 and 8, respectively. The number of surface ligand molecules varies from chain to chain along the Rows. As a rule, each of the superficial sites has many vacancies within each protein Row. As in 7Hyp/ANS, there is no protein chain with a ligand molecule bound in each of its superficial sites. The frequency of interstitial site population increases from site 4 (occupied in 13 chains) to site 8 (29 chains).

**Figure 10:**
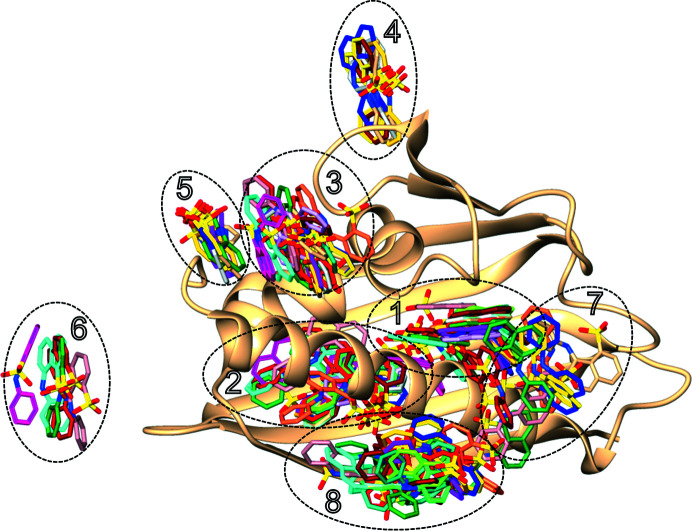
Superposition of all ANS molecules onto a representative Hyp-1 chain *L*. The main binding sites located in two internal chambers (1 and 2) and in a deep surface pocket (3) display better ligand conformational stability than the interstitial sites 4–8. The color code of the ANS molecules corresponds to the color scheme used for the protein chains in Fig. 5 ▸.

Superposition of all of the ANS molecules onto one common C^α^ frame of all of the Hyp-1 chains reveals significant stability of the ligand positions in sites 1, 2, 3, 4 and 5 (Fig. 10 ▸). For sites 6, 7 and 8 a higher positional and conformational variability is shown as an imperfect overlap of the individual molecules, while their location with regard to the respective secondary-structure elements is preserved.

#### 3.8.2. Conformation of the ANS molecules   

The geometry of the ANS molecules was analyzed by examination of three dihedral angles, τ_1_ (C2—C1—S—O), τ_2_ (C7—C8—N—C11) and τ_3_ (C8—N—C11—C), which describe the orientation of the sulfonate group and of the aniline substituent relative to the naphthalene plane, and the orientation of the phenyl ring of the aniline moiety, respectively. The ANS atom numbering scheme is based on IUPAC recommendations and the system adopted in 7Hyp/ANS (Sliwiak *et al.*, 2015 ▸). The conformations of the ANS molecules expressed by the torsion angles τ_1_, τ_2_ and τ_3_ depend on the binding sites, which enforce local ligand deformations. Typically, the ANS molecules are characterized by significant rotational variability of the sulfonate group, with τ_1_ close to 0° owing to the trigonal geometry of the *R*SO_3_^−^ anion. The orientation of the aniline moiety relative to the naphthalene aromatic system seems to be well preserved regardless of the ANS binding site. The values of the torsion angle τ_3_ are close to 0°, which contradicts the geometry of the reference AMANNS structure (∼27°), although the values of the remaining τ_1_ and τ_2_ angles correspond quite well to the reference ligand geometry (Table 4 ▸).

### 3.9. Ligand identification in the electron density   

The presence of a large hydrophobic cavity (Fernandes *et al.*, 2013 ▸) in many PR-10 proteins strongly suggests a bio­logical role in binding specific small-molecule ligands. The most promising candidates for PR-10 cargoes are phytohormones (Fernandes *et al.*, 2013 ▸), with confirmed molecules representing cytokinins (Pasternak *et al.*, 2006 ▸; Fernandes *et al.*, 2008 ▸, 2009 ▸; Kofler *et al.*, 2012 ▸; Ruszkowski *et al.*, 2013 ▸; Sliwiak *et al.*, 2016 ▸), gibberellins (Ruszkowski *et al.*, 2014 ▸), brassino­steroids and their derivatives (Marković-Housley *et al.*, 2003 ▸), and abscisic acid (Sheard & Zheng, 2009 ▸). Another exciting candidate is melatonin, which has only recently been recognized as a plant hormone (Arnao & Hernández-Ruiz, 2018 ▸), especially in view of its antioxidant properties (Arnao & Hernández-Ruiz, 2015 ▸) and its documented ability to mitigate cross-talk between other plant mediators (Erland *et al.*, 2018 ▸). Recently, a possible mechanism for this cross-talk has been illuminated by structural studies (Sliwiak *et al.*, 2018 ▸). The fact that PR-10 proteins are specific melatonin binders is now beyond doubt thanks to crystallographic studies, which include the structure of a Hyp-1 complex (Sliwiak *et al.*, 2016 ▸) that confirmed binding sites 1, 2 and 3, which were previously known only from the 7Hyp/ANS complex. However, the 7Hyp/ANS structure revealed that unlike the MEL ligand, ANS is also able to bind the protein at superficial sites. This fact could explain why even at high concentrations MEL is unable to displace ANS from the protein during ADA assays, as manifested by a strong ANS fluorescence signal under all assay conditions (Sliwiak *et al.*, 2015 ▸). To assess possible competition between ANS and MEL for Hyp-1 internal binding sites, in the present project we have attempted crystallization of the protein in the presence of a mixture of both ligands. As described above, the result was another modulated crystal structure of a complex with the predominant presence of the ANS ligand, which suggests that this artificial molecule is a better Hyp-1 binder than the natural hormone melatonin. It should be stressed, however, that in our experiments it was not possible to obtain Hyp-1–ANS crystals by co-crystallization at MEL:ANS ratios of higher than 1:2 (for example 1:1, 2:1 and 3:1), which provides indirect evidence of MEL competition. In other words, we can only conclude from the present structure that ANS cannot be displaced by MEL when the ANS concentration is at least twofold higher than the MEL concentration in co-crystallization conditions.

Nevertheless, among the numerous (178) ligand molecules in this 9Hyp/ANS structure, there is at least one site, *V*(7), that is suggestive of melatonin binding. The mechanism of blocking the internal MEL binding sites with ANS has already been observed in the 7Hyp/ANS complex structure. Comparison of that structure with the present results additionally suggests the strong conservation of the superficial ANS binding sites and the inability of MEL to replace ANS as a networking glue.

**Figure 11:**
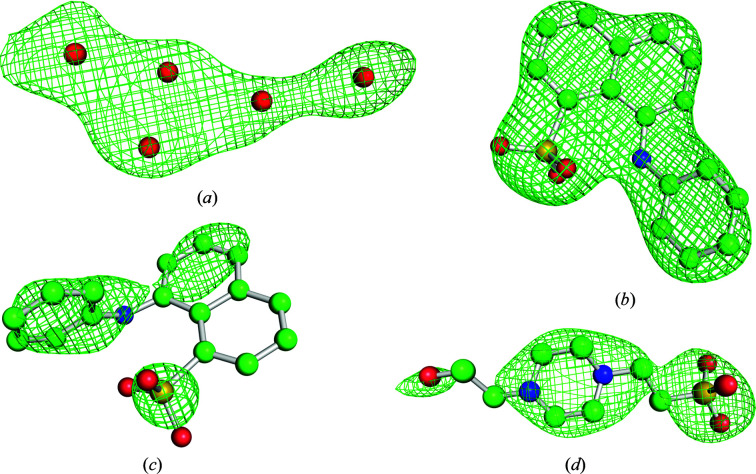
*mF*_o_ − *DF*_c_ polder OMIT maps contoured at 3.0σ for selected 9Hyp/ANS ligands. (*a*) The mysterious electron-density patch at site *V*(7) tentatively interpreted as MEL but ultimately marked with five water molecules (red spheres). (*b*) ANS at site *P*(1) with good definition in the electron density. (*c*) A poorly defined ANS molecule at *S*(2). (*d*) The single HEPES molecule at site *P*(7).

The electron density in the *V*(7) site is compatible in shape with a MEL ligand, especially with its flat indole ring. Attempts to model an ANS molecule or a superposition of MEL and ANS at this site were unsuccessful, leading to deterioration of the refinement parameters and the electron density. Nevertheless, a single fully occupied melatonin molecule was tentatively modeled in this area and this interpretation was validated using polder OMIT maps (Liebschner *et al.*, 2017 ▸) generated in the *Phenix* package. Briefly, a significant advantage of this method over the commonly used OMIT maps is bulk-solvent exclusion from the selected OMIT area and improvement of the resulting map interpretation. A detailed analysis of the MEL polder maps revealed that the electron density assigned to MEL is likely to show bulk solvent or noise rather than a ligand. In this context, the presence of a single MEL molecule at this site has been concluded to not be sufficiently supported by experimental evidence. As shown in Fig. 11 ▸(*a*), because of uncertain interpretation, this region was marked with several dummy water molecules labeled UNL.

In parallel tests, polder map analysis confirmed the presence of ANS in positions with well defined electron density (Fig. 11 ▸ *b*) as well as in some more problematic and difficult-to-interpret positions (Fig. 11 ▸ *c*). Polder OMIT maps also confirmed the correct modeling of the single HEPES molecule at the *P*(7) binding site (Fig. 11 ▸ *d*) and an artifactual citrate (FLC) molecule from the crystallization solution.

Although there is no clear evidence for the presence of MEL in the electron density, the change in crystal packing and in ANS saturation in the present structure in comparison with the crystal obtained by ‘pure’ ANS co-crystallization (Sliwiak *et al.*, 2015 ▸) is an indirect indication of the effect of melatonin on the co-crystallization process. The ANS ligands in the previous 7Hyp/ANS structure were all (with one exception at 0.5) at full occupancy in their electron density and there was not even a trace of residual ANS electron densities in the empty sites. In the present structure, we observe that as many as 50 of 156 ANS molecules (Fig. 9 ▸) have partial occupancy. The comparable resolution of the two structures (2.4 *versus* 2.3 Å) cannot explain this situation. The presence of MEL in the present experiment could weaken the ANS binding, leading to fractional occupancy in some cases. Moreover, even mild competition between MEL and ANS for the internal binding sites could increase the effective ‘pool’ of ANS available for intermolecular binding, thus increasing the molecular-glue potential of ANS.

### 3.10. Protein hydration   

With only a slight increase in resolution (from 2.4 to 2.3 Å), the present model contains 152 well defined water molecules, which is significantly more than in the 7Hyp/ANS structure (35), at least far beyond the 9/7 proportion. Nevertheless, in view of the immense dimensions of the unit cell and with a Matthews volume of 3.12 Å^3^ Da^−1^, corresponding to 60.6% solvent, one would expect many more solvent molecules, perhaps of the order of 4000. The small number of water molecules identified in the electron density is most certainly the effect of structural disorder (nonperiodicity) introduced by the superstructure modulation.

Nearly half of the water molecules are found trapped in chamber 1 of the binding cavity, forming hydrogen bonds to the N^η ^atom of Arg27 (in 11 out of 36 protein molecules), the N^ζ^ atom of Lys139 (eight cases), the N^δ1^ or N^∊2^ atoms of His70 (six cases) or the OH group of Tyr84 or Tyr101 (four cases). A similar situation was noted in 7Hyp/ANS, where five of the 35 confidently identified water molecules occupied positions inside the internal chamber 1, stabilized by interactions with the N^∊2^ atoms of His70 and Gln146, with eight more water molecules found at the E1 entrance. This suggests a stabilizing function of water molecules within the hollow core of Hyp-1. In 9Hyp/ANS, five water molecules mediate protein–ligand contacts, typically at ANS binding site 1. These interactions include hydrogen bonds between the O atoms of the ANS sulfonate group and the side chains of Tyr101 or Arg27. Some water molecules are found near entrance E2, between strands β1 and β7, where ten of them serve as binding mediators between the ANS molecule at site 2 and the O^∊2^ atom of Glu148. In this role, the water molecules may be viewed as a lubricant facilitating filling of the hydrophobic protein core.

Interestingly, no water molecules were found inside the second internal cavity chamber (site 2), despite the presence of residues such as the charged Lys8 and Arg27 or the polar Tyr141 and Tyr144 that are capable of hydrogen-bond formation. This is analogous to the situation in 7Hyp/ANS, where five water molecules were found close to entrance E2 but none were found within the spacious volume of the internal chamber 2. Generally, surface water molecules occupy positions on the protein surface near helix α3, forming hydrogen bonds to the N^∊2^ atom of Gln146 (five cases) or the O^∊2^ atom of Glu142 (six cases). Solitary water molecules linked to the N^∊2^ or N^δ1^ atoms of the histidine ring were found at helix α1 (His17), helix α2 (His28) and loop L5 (His63). A small number of isolated bulk-solvent water molecules with excellent electron density were retained in the model despite having no obvious inclusion in the hydrogen-bonding network of the hydration system.

### 3.11. Crystallization artifacts in the 9Hyp/ANS structure   

In addition to 156 ANS molecules, a poorly confirmed potential MEL ligand and 152 water molecules, the 9Hyp/ANS crystal structure also contains other ligands interacting with the protein at the canonical binding sites 1–8. Of the ten modeled sulfate anions, two occupy binding site 2, where they are stabilized, analogously to ANS, by hydrogen bonds to the N^ζ^ atom of Lys8, strongly suggesting a vestigial character of the ANS sulfonate group, which is visible (thanks to its strong electron count and specific anchoring) despite the disorder of the remaining part of the ligand. This hypothesis is supported by positive electron-density peaks in the vicinity of these sulfate anions, even if it was not possible to reliably interpret them as complete ANS molecules. The remaining eight sulfate anions are located on the protein surface in areas normally reserved for ANS binding sites 8 (five ions) and 7 (three ions). Typically, their position is stabilized by direct hydrogen bonds to the positively charged N atoms of the Lys138 and Lys139 side chains. Interesingly, in five cases a fully occupied ANS molecule was found at a distance of less than 5 Å from the sulfate anions, without any direct interactions.

A detailed examination of the difference electron-density maps near the end of the refinement revealed the positions of seven citrate anions (FLC) from the precipitant. The FLC molecules were found only at ligand-binding sites 7 and 8, making direct hydrogen-bond contacts with the N^η1^ atom of Arg93 (at binding site 7) or the N^ζ^ atom of Lys134 (site 8).

Dimethyl sulfoxide (DMS) molecules from the stock solution of ANS were modeled in the area of binding sites 5 (chain *N*) and 8 (chains *G *and *V*). In contrast to the two interstitial DMS molecules placed between chains *N* and *V*, the remaining single DMS molecules are found in the vicinity of fully occupied ANS molecules. A molecule of the buffer compound HEPES (EPE), also containing a sulfonate group, displaced ANS at binding site 7 of chain *P*. Its docking is stabilized via an interaction with the side chain of Lys138. The shape of the electron density corresponds to a puckered piperazine ring in a chair conformation, despite gaps at the hydroxyethyl and ethanesulfonate chains. The sulfonate group of the HEPES molecule mimics the interaction of the same group of ANS at this site.

## 4. Conclusions   

Previous studies of Hyp-1, a PR-10 protein from *H. perforatum*, revealed that in complex with the fluorescent dye ANS (8-anilinonaphthalene-1-sulfonate) it forms tetartohedrally twinned crystals (7Hyp/ANS) with sevenfold commensurate modulation along the **c** direction of the *C*2 supercell. Pre-incubation of the Hyp-1 protein with an ANS–melatonin mixture and further crystallization trials using the final crystal-growth conditions for the 7Hyp/ANS complex resulted in a different tetartohedrally twinned *C*2 crystal form (9Hyp/ANS) with ninefold noncrystallographic repetition of the same structural motif (two Hyp-1 dimers) along **c** as well as non­crystallographic translational perturbations along the **a** and **b** directions. A physical manifestation of this superstructure modulation is the fluctuation of the diffraction-pattern intensity, with strong reflections for the *l* index of 9*n* and also in between at *l* = 9*n* ± 4. In structural terms, interpretation of this crystal structure as commensurately modulated requires as many as 36 protein molecules in the asymmetric part of an expanded unit cell (supercell) for its description. The present 9Hyp/ANS structure with ninefold modulation was solved by MR using a modified *Phaser* algorithm that takes account of the effects of translational and rotational noncrystallographic symmetry, using the previous 7Hyp/ANS model as a probe. The S(*H*) and *L*-tests suggested at least partial crystal twinning, despite the fact that the detection of twinning is obliterated by translational NCS (tNCS). The twinning was ultimately confirmed (with the four twinning fractions refined at ∼0.25) by successful refinement of the superstructure with *REFMAC*, which converged with an *R* and *R*_free_ of 0.226 and 0.257, respectively, for the diffraction data extending to 2.3 Å resolution. Each Hyp-1 molecule harbors three internal ligand-binding sites, which are variously populated by 95 ANS ligands in the 36 copies of the protein. In addition, there are 61 ANS molecules bound on the surface of the Hyp-1 molecules, which are most likely to be the generator of superstructure modulation. Furthermore, other buffer-solution molecules (sulfate, HEPES, citrate and dimethyl sulfoxide) were found in the structure at selected ligand-binding sites that are typically reserved for ANS. This phenomenon of ligand substitution at what appears to be a random selection of binding sites has not been observed in Hyp-1 complexes before. Although the presence of the single melatonin molecule (which was the ligand of choice for this crystallization experiment) proved to be impossible to maintain after analysis using polder maps, this mysterious area of electron density, which was pragmatically modeled as an unidentified ligand marked by several water molecules, may correspond after all to a poorly ordered or partially degraded melatonin molecule, although its location in binding site 7 does not correspond to the melatonin binding sites (1, 2 and 3) known from a Hyp-1–MEL complex structure, but rather overlaps with a typical interstitial ANS site. Since in the present study slightly modified crystallization conditions (the addition of melatonin to the incubation mixture with ANS) triggered a new type of structure modulation, it is quite possible that other superstructure modulation variants of Hyp-1 complexes could be engineered using different melatonin (or other additive) concentrations.

### 4.1. Problems with Protein Data Bank deposition   

Atomic coordinates and structure factors have been deposited in the Protein Data Bank (PDB) under the accession code 6sjj. Unfortunately, owing to the gargantuan size of this crystal structure (36 protein molecules labeled using both upper-case and lower-case characters) the PDB validation system was unable to produce a sensible validation report, most likely because of an internal confusion between small and capital chain identifiers. For instance, the official validation report contains an enormous list of bogus short contacts, many of them corresponding to absurd distances such as 181.14 Å. We have inspected those ‘short distances’ very carefully and confirmed their spurious character. Since this deposition problem could not be resolved over a long time, we offer an old-fashioned solution, namely that the atomic coordinates and structure factors are available directly from the authors upon request. The raw X-ray diffraction images have been deposited in the RepOD repository at the Interdisciplinary Centre for Mathematical and Computational Modeling (ICM) of the University of Warsaw, Poland and can be downloaded using the following digital object identifier (DOI): https://doi.org/10.18150/repod.6735329.

## Supplementary Material

PDB reference: Hyp-1–ANS complex with ninefold structure modulation, 6sjj

Raw diffraction images.: https://doi.org/10.18150/repod.6735329

## Figures and Tables

**Table 1:** Data-collection statistics Values in parentheses are for the highest resolution shell.

Beamline	14.2, BESSY II
Wavelength (Å)	0.9184
Temperature (K)	100
Cryoprotectant	1.4 *M* sodium citrate
Crystal dimensions (mm)	0.4 × 0.2 × 0.2
Space group	*C*2
*a*, *b*, *c* (Å)	148.85, 148.85, 385.40
α, β, γ (°)	90.00, 90.00, 90.00
Resolution (Å)	31.58–2.30 (2.39–2.30)
No. of reflections (measured/unique)	355613/127324
Completeness (%)	99.3 (94.2)
Multiplicity	2.8 (2.6)
〈*I*/σ(*I*)〉	6.22 (1.28)
CC_1/2_	0.978 (0.620)
*R*_merge_ †	0.066 (0.691)

† *R*_merge_ = 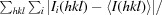 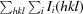, where 〈*I*(*hkl*)〉 is the average intensity of reflection *hkl*.

**Table 2:** Refinement statistics

Refinement program	*REFMAC*5
Resolution (Å)	31.58–2.30
No. of working/test reflections	123350/3974
*R*_work_/*R*_free_ †	0.226/0.257
Protein/ANS molecules in asymmetric unit	36/156
No. of atoms	
Protein	44814
Ligand	3276
Water	152
Other	176
〈*B*〉 (Å^2^)	
Protein	63.2
Ligand	58.3
Water	58.3
Other	56.3
R.m.s.d. from ideal	
Bond lengths (Å)	0.013
Bond angles (°)	1.72
Ramachandran statistics‡ (%)	
Favored	92.6
Allowed	6.9
Outliers	0.5
PDB code	6sjj

† *R* = 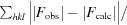 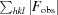, where *F*_obs_ and *F*_calc_ are the observed and calculated structure factors, respectively. *R*_free_ is calculated analogously for the test reflections, which were randomly selected in thin 2θ shells and excluded from the refinement.

‡ Assessed with *MolProbity* (Chen *et al.*, 2010 ▸).

**Table 3:** Comparison of protein–ligand interactions (<3.2 Å) for both the 9Hyp/ANS and 7Hyp/ANS modulated crystal structures at internal binding sites 1, 2 and 3 Residues highlighted in bold form hydrogen bonds to O atoms of the ANS sulfonate group. For each site, the residues are listed in decreasing order of contact number.

Binding site (No. of chains with ANS at this location in 9Hyp/ANS|7Hyp/ANS)	Residues involved in contact with ANS	
	9Hyp/ANS	7Hyp/ANS
1 (36|23)	**Arg27**, Gln35, Leu31, Val91, Lys139, Ala140, Glu132, Gly136	**Arg27**, Gln35, Leu31, Val91, Glu132, Gly132, Lys139, ANS at site 7
2 (31|24)	**Tyr144**, **Tyr141**, **Ala140**, Tyr84, Lys8, Glu10, Leu19, Ile116, Arg27	Tyr144, **Lys8**, Leu19, Ile116, **Glu10**, Tyr141, Leu23, Arg27, Tyr84
3 (28|13)	**Tyr150**, **Lys33**, Phe158, Val30, Val157, Ala22, Val147	**Tyr150**, **Lys33**, Val30, Val147, Phe158, Val157, Leu151

**Table 4:** ANS conformation described by the mean values (and their standard deviations) of the τ_1_, τ_2_ and τ_3_ torsion angles (°) for ligands grouped in the respective binding sites As a reference, the AMANNS model structure (Weber & Tulinsky, 1980 ▸) from the CSD database was used. Interstitial binding sites were bundled into two groups: ligands 4, 5 and 6 are responsible for joining three adjacent Hyp-1 chains, meaning that a given ANS molecule may be labeled 4, 5 or 6, depending on the nearest Hyp-1 site with which it interacts. Analogously, sites 7 and 8 were merged because they glue together two protein chains. For comparison, results from the 7Hyp/ANS study are given in italics below the 9Hyp/ANS data. ANS atom numbering follows IUPAC recommendations and the convention adopted for 7Hyp/ANS (Sliwiak *et al.*, 2015 ▸).

	Site					
Torsion angle	1	2	3	4/5/6	7/8	AMANNS
C2—C1—S—O† (τ_1_)	−2 (2)	0 (2)	−1 (2)	−2 (3)	2 (10)	1
	*3 (2)*	*1 (2)*	*2 (2)*	*−5 (2)*	*−1 (2)*	
C7—C8—N—C11 (τ_2_)	48 (7)	32 (4)	20 (8)	28 (10)	18 (6)	42
	*38 (2)*	*12 (1)*	*−2 (6)*	*−21 (5)*	*11 (6)*	
C8—N—C11—C‡ (τ_3_)	1 (1)	1 (1)	−1 (1)	−1 (2)	2 (1)	27
	*11 (3)*	*24 (5)*	*−3 (11)*	*−31 (4)*	*1 (12)*	

† Sulfonate O atom that minimizes |τ_1_|.

‡ Aniline C atom that minimizes |τ_3_|.
